# Transcriptional Up-Regulation of APE1/Ref-1 in Hepatic Tumor: Role in Hepatocytes Resistance to Oxidative Stress and Apoptosis

**DOI:** 10.1371/journal.pone.0143289

**Published:** 2015-12-01

**Authors:** Vittorio Di Maso, María Gabriela Mediavilla, Carlo Vascotto, Francesco Lupo, Umberto Baccarani, Claudio Avellini, Gianluca Tell, Claudio Tiribelli, Lory Saveria Crocè

**Affiliations:** 1 FIF- Fondazione Italiana Fegato Area Science Park Basovizza. Trieste, Italia; 2 Dipartimento Scienze Mediche e Biologiche, Università di Udine. Udine, Itala; 3 Chirurgia Generale 2 Centro Trapianto Fegato Universitá Torino. Torino, Italia; 4 Dipartimento Scienze Mediche, Università di Trieste. Trieste, Italia; University of Navarra School of Medicine and Center for Applied Medical Research (CIMA), SPAIN

## Abstract

**Objective:**

Human Hepatocellular Carcinoma (HCC) is the fifth most frequent neoplasm worldwide and the most serious complication of long-standing chronic liver diseases (CLD). Its development is associated with chronic inflammation and sustained oxidative stress. Deregulation of apurinic apyrimidinic endonuclease 1/redox effector factor 1 (APE1/Ref-1), a master regulator of cellular response to oxidative stress, has been associated with poor prognosis in several cancers including HCC.

**Design:**

In the present study we investigated the APE1/Ref-1 mRNA levels in cirrhotic and HCC tissues obtained during HCC resection. The possible protective role of APE1/Ref-1 against oxidative stress and apoptosis was evaluated *in vitro* in immortalized human hepatocytes (IHH) over-expressing APE1/Ref-1.

**Results:**

APE1/Ref-1 was up-regulated in HCC, regulation occurring at the transcriptional level. APE1/Ref-1 mRNA content increased with the progression of liver disease with the transcriptional up-regulation present in cirrhosis significantly increased in HCC. The up-regulation was higher in the less differentiated cancers. *In vitro*, over-expression of APE1/Ref-1 in normal hepatocytes conferred cell protection against oxidative stress and it was associated with BAX inhibition and escape from apoptosis.

**Conclusion:**

APE1/Ref-1 is up-regulated in HCC and this over-expression correlates with cancer aggressiveness. The up-regulation occurs at the transcriptional level and it is present in the earliest phases of hepatocarcinogenesis. The APE-1/Ref-1 over-expression is associated with hepatocyte survival and inhibits BAX activation and apoptosis. These data suggest a possible role of APE1/Ref-1 over-expression both in hepatocyte survival and HCC development calling attention to this molecule as a promising marker for HCC diagnosis and treatment.

## Introduction

Human Hepatocellular Carcinoma (HCC) is the most common malignant primary liver tumor [1], the most serious complication of long-standing chronic liver disease (CLD) and the third cause of cancer-estimated deaths worldwide [2], symptomatic only at the advanced stage when effective and radical therapies are limited. Thus, a better understanding of the molecular events inducing hepatocyte transformation is of the utmost importance.

Hepatocarcinogenesis is a multistep process in cirrhosis [3]. Hepatocyte neoplastic transformation is associated with hepatocyte proliferation [4], activation of oncogenes, DNA rearrangement, chromosomal instability [5], and mitochondrial DNA damage [6]. Thus, selected cell population proliferate with activation of survival pathways inducing malignant phenotype [7,8] as in the case of apoptotic pathway deregulation [4,9]. CLD are all characterized by chronic inflammation and increased production of free radicals [10,11]. Oxidative stress leads to cellular damage and cell function disruption causing hepatocyte death and regeneration [11] therefore increasing liver cell turnover in a context of chronic inflammation and oxidative damage [12].

APE1/Ref-1 is a master regulator of cellular response to oxidative stress, involved in transcriptional regulation of gene expression during adaptive cellular response to oxidative stress and in base excision repair pathway of oxidative DNA lesions [13]. APE1/Ref-1 is regulated at both the transcriptional and post-translational levels; Reactive Oxygen Species (ROS) induce APE1/Ref-1 expression [14,15].

In a previous work we have addressed the functional protective role of APE1/Ref-1 in preventing cell death upon genotoxic treatment and Fatty Acid accumulation using hepatic cell lines [16]. Using both the APE1/Ref-1 redox inhibitor (E3330) and APE1/Ref-1 functional mutants expressing clones, we were able to find that APE1/Ref-1 overexpression protects cells toward different genotoxicants (i.e. H_2_O_2_, methyl methanesulfonate and etoposide). Moreover, treatment with the E3330 prevented the functional activation of NF-κB *via* the alteration of APE1/Ref-1 sub-cellular trafficking and reduced IL-6 and IL-8 expression induced by TNF-α and FAS accumulation through blockage of the redox-mediated activation of NF-κB. Therefore, APE1/Ref-1 overexpression observed in hepatic cancer cells may reflect an adaptive response to cell damage and may be responsible for further cell resistance to chemotherapy and for the onset of the inflammatory response.

Oxidative damage play a role in inflammation driven carcinogenesis [17,18], as in the case of HCC, it facilitates tumorigenesis in several ways [19,20] by deregulation of preneoplastic and neoplastic cell apoptosis [21,22], developing resistance against cell death signaling pathways. Alteration of APE1/Ref-1 intracellular distribution pattern and its up-regulation have been shown to correlate with clinical outcome in different human cancers [23,24]. We have previously demonstrated that in HCC, APE1/Ref-1 sub-cellular localization have a prognostic significance being its cytoplasmic localization associated with a worst prognosis [25]. However, no data are available about *APEX1* gene expression level in human HCC and cirrhotic tissues with respect to normal liver.

In this work, the mRNA expression level of APE1/Ref-1 has been studied both *in vivo* and *in vitro* to investigate the possible association between APE1/Ref-1 and HCC development. We also investigated the APE1/Ref-1 protective role *in vitro* by evaluating hepatocyte response to oxidative stress and Bax activation and apoptosis induction after APE1/Ref-1 over-expression.

## Materials and Methods

### Patients

Nineteen HCC subjects were consecutively enrolled and treated, six with liver transplantation (OLT) and 13 with liver resection. One liver donor was used as normal control (NL). Sixteen of them were males and three were females with M/F ratio of 5/1 and a mean age of 63±7 years (no differences between sexes). Eight patients were HCV, 5 HBV and 6 alcohol abusers.

Samples of HCC and distal liver cirrhosis (DLC) were obtained at the time of surgery. Formalin fixed and paraffin embedded, 4-μm sections were cut and stained with hematoxylin-eosin. Cirrhosis was clinically staged by Child-Pugh score [26,27] 12 being A, 6 B and 1 C. Tumor staging was assessed using BCLC [27], three were A1, 3 A4, 13 A3. HCC was classified according to Edmondson and Steiner criteria [28] 14 being G1-G2 and 5 G3-G4, analyzed by a single operator. For molecular analysis of *APEX1* expression, samples of DLC and HCC collected during the surgery were immediately snap frozen and stored at -80°C until the RNA extraction. All clinical data have been collected at the time of surgery. For each patient a written informed consent form has been obtained. Study was approved by Comitato Indipendente per la Bioetica (C.I.B.), I.R.C.C.S. Trieste, Prot CE/V-55, (11/5/2005)

### Cell lines

APE1/Ref-1 expression pattern was evaluated using Immortalized Human Hepatocytes (IHH) [29] (non tumoral cells), HuH-7 (well-differentiated hepatoma) and JHH-6 (poor-differentiated hepatoma) cell lines. IHH were grown in DMEM/F12 supplemented by Fetal Bovine Serum (FBS) 10%, Dexamethasone 10^−6^ M, bovine insulin 10^−8^ M. HuH-7 (JCRB0403) and JHH6 (JCRB1030) have been purchased from Japan Health Science Research Resources Bank Celland were grown respectively in Dulbecco's modified Eagle's and Williams'E medium supplemented by FBS 10% and L-Glutamine 2 mM, and used within 20 passage numbers.

### Transfections

All transfections were carried out in IHH cells. The sequences of interest were cloned into p3XFLAG-CMV^™^-14 obtaining 2 different expression vectors with the Flag at the C-terminus: 1) *p3XFLAG* empty vector used as control (IHH/p3X), 2) *pRef-1* containing the cDNA coding for the wild type protein (IHH/pRef-1). Transfection was performed using Lipofectamine 2000 according to the manufacturer’s instructions. Selection of stable episomal transfectants has been obtained using G418 antibiotic.

### Real Time RT-PCR

Total RNA was extracted using Tri-Reagent kit in accordance with manufacturer’s instructions. Quantification and quality evaluation were performed spectrophotometrically being RNA quality criteria sufficed when A260/A280 ratio was between 1.8 and 2.0 and A260/A230 ratio greater than 2.0. Total RNA was retrotranscribed using iScript^*™*^ cDNA Synthesis kit and Real-time quantitative PCR was performed according to the iQ^*™*^ SYBR^®^ Green Supermix protocol; 18S and β-actin were used to normalize levels of specific mRNA between samples. Primer pairs were designed using Beacon Designer 6.0 and synthesized by Sigma Genosys. Primer sequences: APE1/Ref-1 (NM_080649) 5’-CTGCCTGGACTCTCTCATCAATAC-3’ and 5’-GAATGCCGTATCCGCTACTCC-3’ 18S (X03205) 5’-TAACCCGTTGAACCCCATT-3’ and 5’-GCGATGATGGCTAACCTACC-3’ β-actin (NM_001101) 5’-CGCCGCCAGCTCACCATG-3’ and 5’-GGCAGAAGGGGAGGTAGCAC-3’. Relative quantification was made using the Pfaffl modification of the ΔCt equation [30,31]. APE1/Ref-1 expression in LC and HCC was expressed relative to NL.

### APE1/Ref-1 protein level analysis *in vitro*

Total protein extraction was obtained using Cell signaling cell Lysis Buffer in accordance with manufacture’s protocol. Western Blot analysis was performed using as primary antibodies anti-APE1/Ref-1 mouse monoclonal antibody (1:200) and anti-α-tubulin (reference protein) monoclonal antibody (Santa Cruz Biotechnology; 1:5000) and Anti-mouse IgG peroxidase conjugated antibody (Dako; 1:2000) as secondary antibody. Western blot was used to quantify the protein amount and comparative analysis of protein level has been performed as previously described [32,32–34], APE1/Ref-1 optical density was normalized to the α-tubulin.

### Nuclear, cytoplasmic and mitochondrial fractionation

Nuclear cytoplasmic and mitochondrial extracts were isolated by differential centrifugation as previously described [35,36]. Cellular purified fractions were separated on a 10% SDS-PAGE gel and evaluated by Western Blot. APE1/Ref-1 was detected with the same antibody and conditions previously described. To test nuclear and cytoplasmic enrichments anti α-tubulin antibody and anti p84 antibody (Abcam Inc., catalog ab487, 1:1000), have been used as cytoplasmic and nuclear markers respectively.

### Immunocytochemistry

IHH, Huh-7 and JHH6 were grown on glass coverslips to reach a 50–60% confluence, fixed with paraformaldehyde 4% and permeabilized with 0.1% Triton X-100, treated with RNAse A and blocked with PBS containing 2.5% FBS. The coverslips were then incubated with the same anti-APE1/Ref-1 primary antibody used for WB, fluorescein isothiocyanate (FITC)-conjugated mouse antibody was used as secondary antibody. Propidium iodide stained the nucleus. Fluorescent images were visualized by Leica DM2000 apparatus, captured by charge-coupled-device camera (Leica DC490) and processed using Adobe Photoshop CS 8.0. Immunocytochemistry was used to access flagged proteins subcellular localization, anti-Flag M2 form Sigma (1:1000) was used to detect flagged APE1/Ref-1, nuclei were stained using Hoechst 33342 and mitochondria were stained using MitoTracker Red CMX ROS.

### Western Blot for APE1/Ref-1 transfected forms

Total protein extracts from each clone were separated on a 10% SDS-PAGE gel as previously described. The production of flagged exogenous APE1/Ref-1 protein form was detected by using anti-Flag M2 antibody Sigma (Sigma, 1:1000). To evaluate the endogenous protein production, APE1/Ref-1 was detected with the anti-APE1/Ref-1 monoclonal antibody.

### Oxidative stress induction and MTT analysis

IHH/pRef-1and IHH/p3X were plated (25000 cells/cm^2^) in 24 well tissue culture plates. After 24 hours cells were exposed to hydrogen peroxide at the following concentrations: 0, 100, 150, 200 and 250 μM for additional 24h, and cytotoxicity was tested by MTT reduction assay. Absorbance was measured in a plate reader (Beckman Coulter LD400) at λ = 562 nm.

### Induction and detection of apoptosis

Apoptosis was induced by irradiating cells (in fixed volumes of culture medium) at 90 mJ/cm^2^ during 12” using a UV Stratalinker 2400 apparatus. To quantify cell death, apoptosis was induced and 6 h after irradiation floating cells and adhering cells were collected and stained with annexin V-FITC and Propidium Iodide (50 μg/mL). Stained cells were then analyzed by flow cytometry using a FACScalibur system (Becton Dickinson). Apoptosis induction was further investigated by evaluating BAX activation and Cytochrome C release by immunocytofluorescence. Cells were stained with mitotracker Red CMX ROS, nuclei were stained by Hoechst 33342. BAX activation was detected using anti-BAX cdc2 antibody (1:200) and Cytochrome C release using anti-Cyt C antibody (1:400) as primary antibodies, anti mouse FITC was used as secondary antibody. Cells were then visualized by Zeiss Axiovert 135TV apparatus; images were captured by Photometric CE200A CCD device camera and processed using Adobe Photoshop CS 8.0. Scoring of BAX Activation and Cytochrome C release was performed by counting at least 100 fluorescent cells per field. Only the cells displaying a clear diffuse cytoplasmic staining for Cytochrome C (cytochrome C release feature) and a clear BAX mitochondrial localization were scored as apoptotic cells.

### Statistical analysis

APE1/Ref-1 mRNA expression levels are expressed as median fold change and interquartile range (IQR) data are analyzed using Wilcoxon matched pairs test (p<0.05). All data obtained *in vitro* represent the mean of at least three different experiments, and data are reported as mean ± Standard Deviation (SD). Comparison between two groups were analyzed via Student’s t test (p<0.05) and data are reported as mean ± Standard Deviation (SD). Comparison between three groups by ANOVA with a Bonferroni posthoc test (p<0.05) and data are reported as mean ± Standard Deviation (SD).

## Results

**Fig 1:**
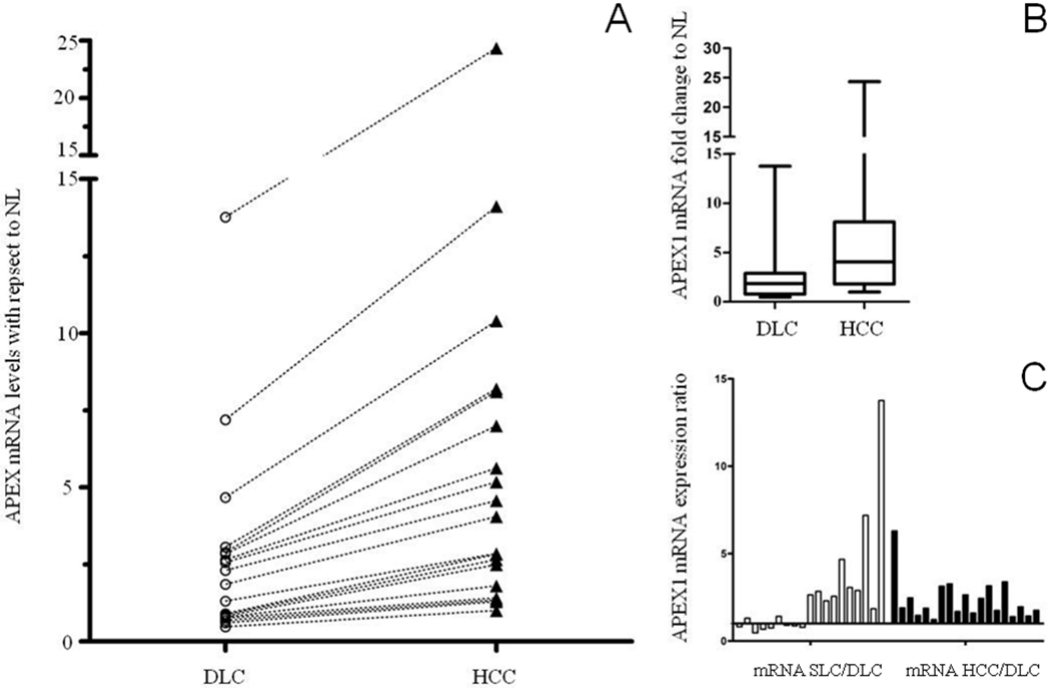
APE1/Ref-1 mRNA relative levels determined by Real Time RT-PCR in HCC affected patients. (A) APE1/Ref-1 mRNA level increase in HCC with respect to DLC for each patient. (B) APEX1 mRNA fold change in HCC with respect to DLC. (* p = 0.002 vs. DLC). (C) SLC/DLC ratio and HCC/DLC ratio for each patient. Abbreviations: DLC: distal liver cirrhosis; SLC: surrounding liver cirrhosis; HCC: hepatocellular carcinoma; NL: normal liver.

APE1/Ref-1 mRNA content was higher in HCC (4.05, IQR 1.8–8.1) than in DLC (1.85, IQR 0.79–2.89) (p = 0.002) (Fig 1B) with increasing levels moving from DLC to HCC (Fig 1A). All showed an up-regulation of APE1/Ref-1 in HCC with HCC/DLC ratio >1 (Fig 1C), while only 57.8% of subjects presented an up-regulation in cirrhosis as compared to NL (Fig 1C) indicating that transcriptional up regulation of *APEX1* gene is always present in HCC and in more than half of cirrhotic samples. Of notice, APE1/Ref-1 up-regulation in HCC differs according to CLD etiology as mRNA levels were significantly higher in those related to HCV (2.11 ± 0.66, p = 0.036) as compared to alcohol (1.16 ± 0.37); intermediate values were found for HBV infection (1.86 ± 0.54). No association has been found between APE1/Ref-1 mRNA level in DLC and HCC tissue with age, sex, Child-Pugh score, HCC grading and HCC staging.

**Fig 2:**
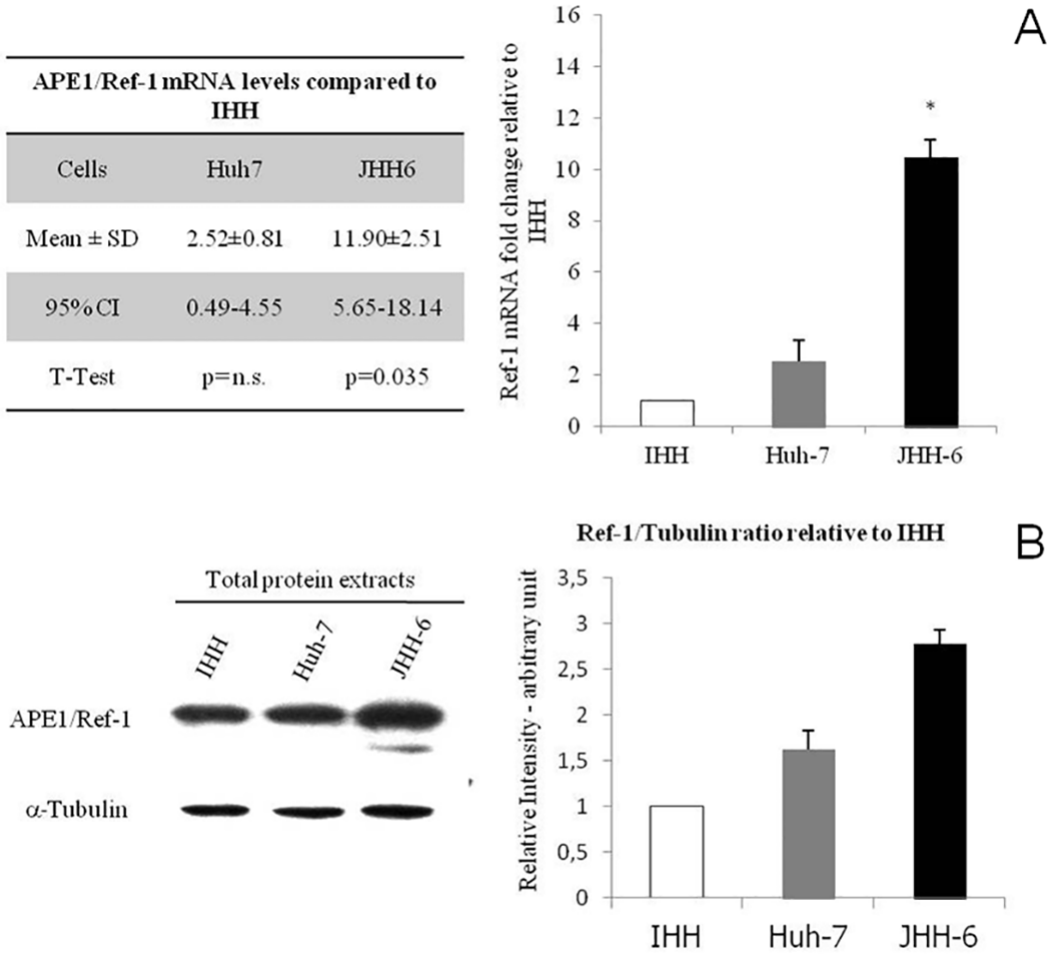
APE1/Ref-1 mRNA and protein levels in Hepatoma cell lines with respect to IHH. (A) Real Time RT-PCR analysis expressed as fold change in mRNA expression of APE1/Ref-1 in hepatoma cell lines as compared to normal hepatocytes (IHH). Data are expressed as mean± SD of five different experiments from 5 different batchs of cells *p = 0.0035 vs. Huh7. (B) WB analysis for APE1/Ref-1 in total extracts of hepatoma cell lines and in normal hepatocytes. Left: representative WB for APE1/Ref-1 detection. Right: band density quantification graph. Samples were normalized to α -tubulin and protein levels in Huh-7 and JHH-6 are relative to IHH cells, data are expressed as mean±SD of five different experiments from 5 different batchs of cells. (* p = 0.001 vs. Huh7).

**Fig 3:**
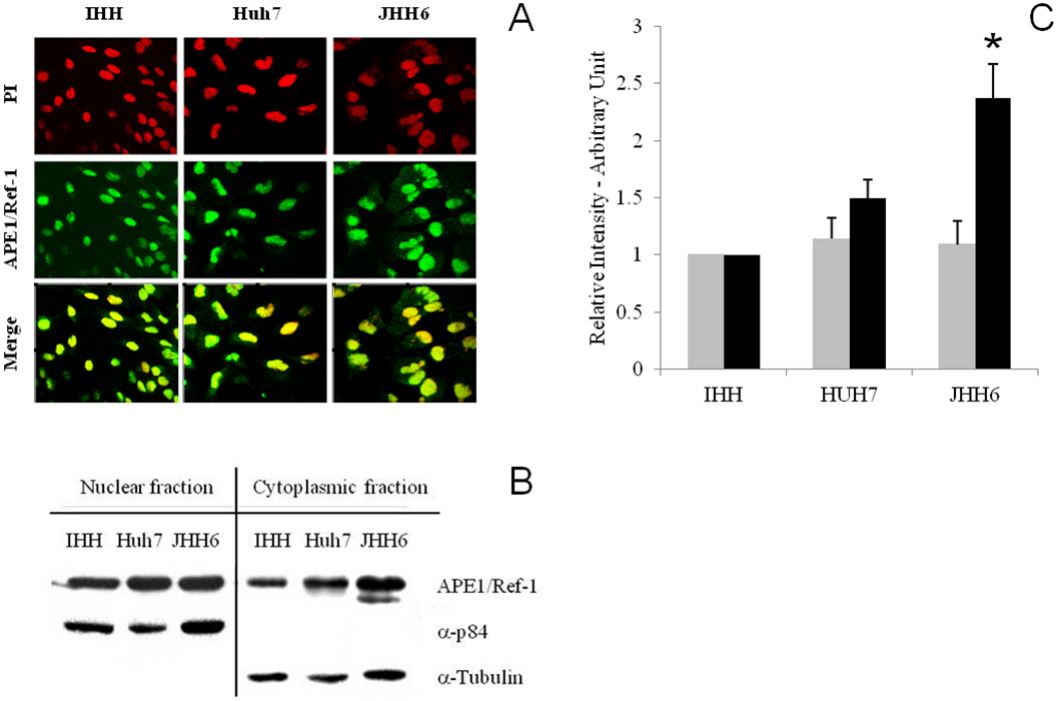
Intracellular localization of APE1/Ref-1 in hepatoma cell lines. (A) Immunofluorescence for APE1/Ref-1 in IHH, Huh-7 and JHH6. In red: propidium iodide (PI) stained nuclei. In green: FITC-immunodetected APE1/Ref-1. APE1/Ref-1 is localized both in the nucleus and in the cytoplasm of each cell line. Merge is obtained overlapping the two images; orange/yellow indicates nuclear localization of APE1/Ref-1 while green indicates cytoplasmic localization of this protein. (B) Western blot analysis for APE1/Ref-1 in IHH, Huh-7 and JHH6 nuclear and cytoplasmic fractions. Representative WB for APE1/Ref-1 in IHH, Huh-7 and JHH6 nuclear and cytoplasmic fractions. (C) Band density quantification graph for nuclear (grey bars) and cytoplasmic fractions (black bars). Samples were normalized to α-tubulin and α-p84 respectively and protein levels in Huh-7 and JHH-6 are relative to IHH cells, data are expressed as mean ± SD of three different experiments from 5 different batches of cells.

APE1/Ref-1 expression *in vitro* demonstrated that APE1/Ref-1 mRNA level was significantly higher (p = 0.0035) in hepatoma cell lines since JHH-6 (11.9 ± 2.51, CI 5.65–18.1) and Huh-7 (2.5 ± 0.81, CI 0.49–4.55) showed respectively a 11.9-fold and 2.5-fold increase in mRNA level with respect to IHH (Fig 2A), with a progressive increase of APE1/Ref-1 gene expression moving from normal hepatocytes to poorly-differentiated cancer cells pointing to an association between APE1/Ref-1 mRNA level and HCC differentiation grading as observed in human tissues. Furthermore, APE1/Ref-1 protein was significantly higher (p = 0.001) in JHH-6 (2.78 ± 0.21) and in Huh-7(1.62 ± 0.15) than in IHH (Fig 2B) indicating that increased mRNA synthesis is associated with increased APE1/Ref-1 protein production especially in poorly-differentiated hepatoma. In addition to the main band that was observed at the expected 37 kDa corresponding to the APE1/Ref-1 full length protein, a second less intense band of a MW around 33 kDa (Fig 2B) was observed only in the poorly-differentiated hepatoma cell lines. This band is consistent with APE1/Ref-1 truncated form lacking the N-terminal 33 amino acids already described [37]. APE1/Ref-1 was found both in the nucleus and cytoplasm (Fig 3A). However, Western blot analysis performed on nuclear and cytoplasmic enriched fractions (Fig 3B) showed higher protein content in JHH6 cytoplasmic fraction with respect to Huh-7 and IHH. On the contrary, the relative APE1/Ref-1 nuclear amount was comparable in the 3 cell lines (Fig 3C). These data confirm the previous observation that APE1/Ref-1cytoplasmic localization is more frequent in poorly differentiated HCC.

**Fig 4:**
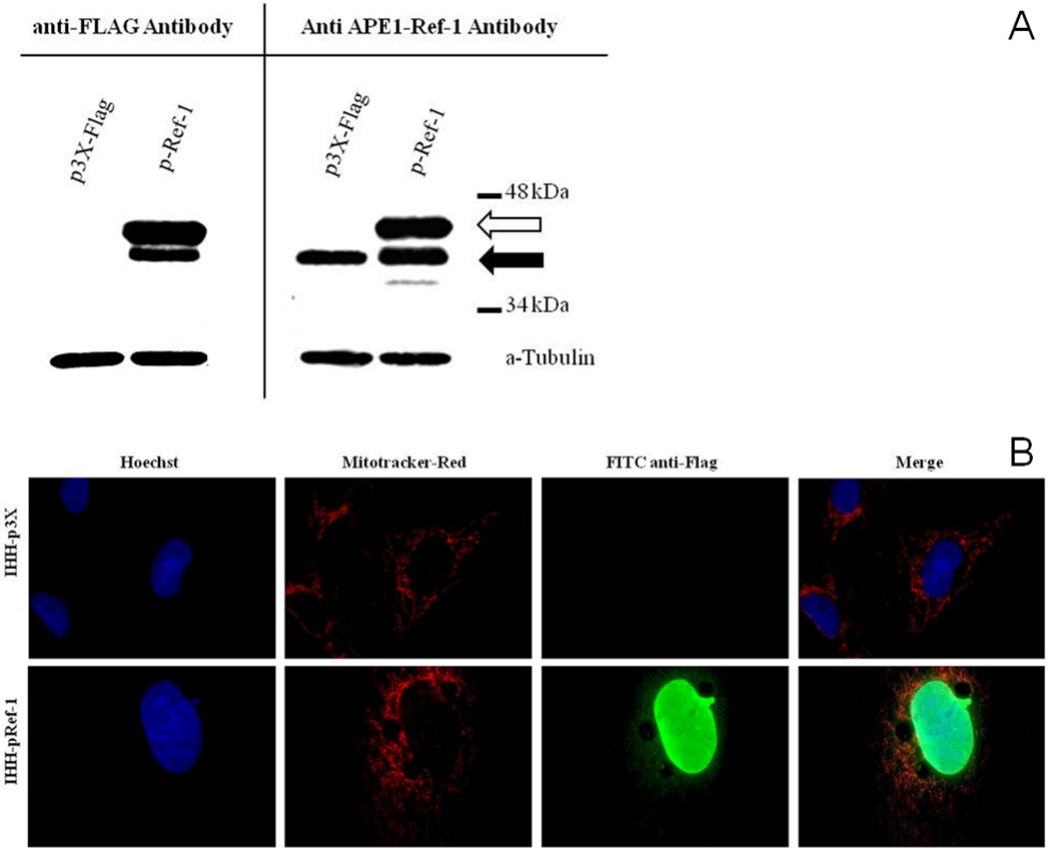
Detection of transfected (exogenous) and endogenous APE1/Ref-1 forms. (A) Western Blot detection of transfected flagged APE1/Ref-1 proteins using anti-Flag antibody (left panel) and detection of endogenous (black arrow) and flagged APE1/Ref-1 (white arrow) using anti-APE1/Ref-1 antibody (right panel). (B) Microphotography of flagged APE1/Ref-1 cellular localization in the two cell lines obtained after transfection. In blue: Hoescht stained nuclei. In red: mitotracker-red stained mitochondria. In green: FITC detected flagged APE1/Ref-1 forms. Merge is obtained overlapping all three images; orange/yellow indicates a mitochondrial localization of the flagged proteins.

**Fig 5:**
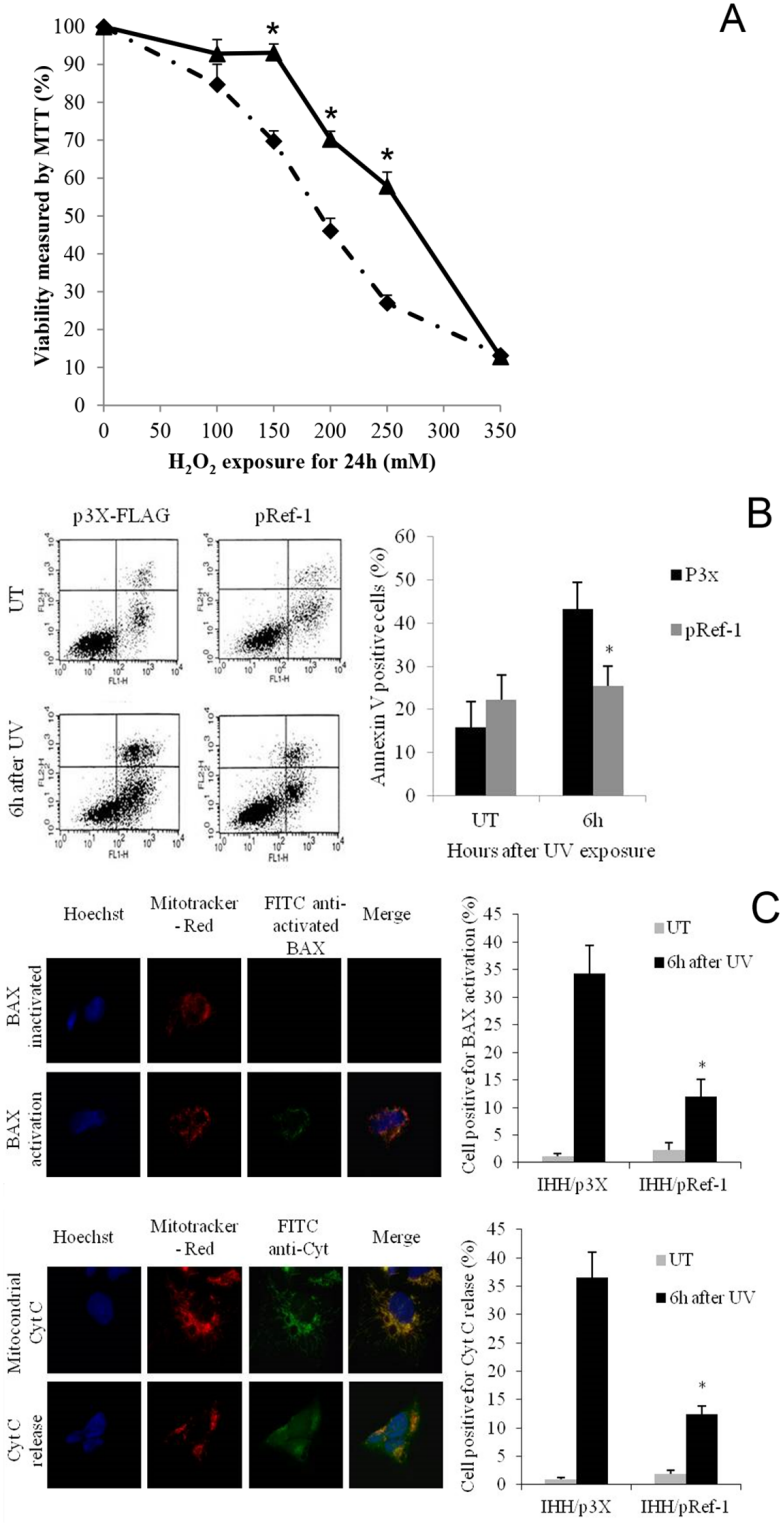
APE1/Ref-1 exerts hepatocytes protection against oxidative insult and apoptosis. (A) Hydrogen peroxide cytotoxicity on transfected IHH measured by MTT assay (black diamond: IHH/p3X; black triangle: IHH/pRef-1). Data are expressed as mean ± SD of three different experiments. (* = different from IHH/p3X; p<0.05). (B) FACS analysis of apoptotic rate after UV irradiation. Cells were treated with UV exposure and after 6 hours FACS analysis for AnnexinV staining was performed to assess the percentage of cells undergoing apoptosis. Left Panel: example of FACS analysis graphs for apoptotic rate at 6 hours after UV irradiation (FL1-H: AnnexinV-FITC; FL2-H: propidium iodide). Right Panel: The bar plot shows the AnnexinV-FITC positive cells mean ± SD of three different experiments from three different batches of cells (black bars: IHH/p3X; grey bars: IHH/pRef-1). (* = different from IHH/p3X). (C) Immunocytochemical detection of Bax activation and Cytochrome C release and positive cell counting. Figures represent an example of immunocytochemical detection of Bax activation (upper figure) and Cytochrome C release (lower figure) after UV treatment. In blue: Hoescht stained nuclei. In red: mitotracker-red stained mitochondria. In green: FITC detected Cytocrome C. Merge is obtained overlapping all three images, orange/yellow indicates a mitochondrial localization of BAX and Cytocrome C. After BAX activation Cytochrome C is released into the cytoplasm losing its mitochondrial localization. Bar graphs represent the percentage of cells with BAX activation and Cytocrome C release after UV irradiation (grey bars: untreated; black bars: 6 hours of UV exposure). Data are reported as mean ± SD of three different experiments. (*p<0.01 vs. IHH/p3X). UT: untreated.

To evaluate the possible role of APE1/Ref-1 on hepatocyte survival, an over-expression strategy was used obtaining IHH/p3X and IHH/pRef-1 cells with preserved hepatocyte cell morphology when compared to IHH parental cells. Both the exogenous flagged APE1/Ref-1 (Fig 4A) and the endogenous protein (Fig 4B) were expressed in the transfected IHH demonstrating that the exogenous APE1/Ref-1 over-expression did not suppress the endogenous protein production. In addition, exogenous APE1/Ref-1 was localized both in IHH nucleus, cytoplasm and mitochondria (Fig 4B) indicating that the intracellular trafficking was not altered either by the FLAG sequence or by the over-expression. When these cells were exposed to oxidative stress, IHH/pRef-1, exerted a significant protection against H_2_O_2_ damage than IHH/p3X, at 150, 200 and 250 μM (p<0.05). At higher concentration (350 μM), this protection disappeared (NS) (Fig 5A). The main difference was found at 250 μM H_2_O_2_ with an IHH/p3X viability almost half of that of IHH/pRef-1 (27.0 ± 2.04 vs. 57.91 ± 3.67, respectively; p = 0.022). These findings collectively indicate, as in other cell lines, that increased expression levels of APE1/Ref-1 protect hepatocytes from oxidative stress damage [38].

Hepatocyte survival ability was studied by inducing apoptosis. Six hours after UV irradiation the number of Annexin V positive cells (Fig 5B) was significantly reduced (p = 0.001) in IHH/pRef-1 than in control cells indicating that APE1/Ref-1 protects hepatocytes from apoptotic stimulus.

Since the inactivation of mitochondrial apoptotic intrinsic pathway is crucial for hepatocyte survival and HCC development, and to further investigate APE1/Ref-1 role in apoptosis escape, we studied BAX activation and Cytochrome C release inhibition. APE1/Ref-1 over-expression significantly inhibited (p = 0.001) BAX activation with a 3 time reduction in IHH/pRef-1 with respect to IHH/p3X (30.8 ± 0.91). This effect was associated with a parallel decrease in Cytochrome C release (p = 0.001) in IHH/pRef-1 (12.4 ± 1.4) with respect to IHH/p3XFLAG (35.5 ± 1.1) (Fig 5C).

## Discussion

APE1/Ref-1 is known to be deregulated in different tumors and this deregulation correlates with tumor aggressiveness and prognosis (39, 40). In HCC, APE1/Ref-1 cytoplasmic localization is more frequent in poorly-differentiated tumors and is associated with a shorter survival time [25]. However, the mechanism linking the cytoplasmic accumulation with hepatocarcinogenesis is still not known. It has been demonstrated that APE1/Ref-1 cytoplasmic localization enhances lung tumor aggressiveness [39]. Hepatocarcinogenesis begins in cirrhotic hepatocytes [40] where persistent cellular damage induces an increased ROS production [41]. APE1/Ref-1 is a master regulator of cellular response to oxidative stress and it has been demonstrated that its intracellular localization has a prognostic significance also in predicting HCC relapse after transplantation [42]. APE1/Ref-1 over-expression depends in part by NF-kB pathway in cancer cells [43]. Nevertheless, no data are available about APE1/Ref-1 gene expression in human HCC tissues. The present study demonstrated, for the first time, that APE1/Ref-1 mRNA synthesis is increased in HCC, suggesting a transcriptional regulatory mechanism of APE1/Ref-1 expression in HCC, in line with the observation of elevated APE1/Ref-1 transcript levels in other tumors such as prostate cancer [44] and melanoma [45]. It has been demonstrated that both APE1/Ref-1 mRNA levels and protein production are altered in chronic viral hepatitis [46], and this agrees with our finding that APE1/Ref-1 is up-regulated mostly in cirrhotic tissue of HCV affected patients. Although in the present study APE1/Ref-1 resulted over-expressed in all HCC specimens, more than half of patients showed an up-regulation also in DLC, suggesting that APE1/Ref-1 transcriptional activation is already present in the earliest phase of liver disease progression, when oxidative burst is elevated and selection of hepatocyte clones able to survive plays a central role in tumor development.

Several evidences demonstrated the involvement of Ape1/Ref-1 over-expression in carcinogenesis. Mi-Hwa Kim demonstrated that APE1/Ref-1 contributes to aggressive colon cancer behavior and functions as an upstream activator in the Jagged1/Notch signaling pathway highlighting its oncogenic effects [47]. Elevated APE1/Ref-1 levels facilitate ROS induced transformation of JB6 cells suggesting that APE1/Ref-1 up-regulation protects cells from oxidative damage facilitating neoplastic transformation [48]. This is important since hepatocarcinogenesis is characterized by a progressive de-differentiation of the hepatocytes [28,28,49,49,50]. This is in line with our finding of a significant increase of APE1/Ref-1 mRNA production from DLC to HCC, pointing to an association between tumor aggressiveness and APE1/Ref-1 up-regulation. The cytoplasmic accumulation of APE1/Ref-1 has been described as a peculiar feature of transformed hepatocytes, and this pattern was described also in many other human cancers [23]. We demonstrated that APE1/Ref-1 protein content was significantly higher in the less differentiated hepatoma cell line that was characterized by a cytoplasmic accumulation of the protein. It has been recently shown that in lung cancer, APE1/Ref-1 cytoplasmic localization is associated with higher tumor aggressiveness and involves NF-kB pathway activation. These findings emphasize the need for a deeper understanding of the mechanism associated with APE1/Ref-1 cytoplasmic accumulation in HCC and the protein functions in this compartment. Whereas APE1/Ref-1 nuclear roles are well established, little is known about APE1/Ref-1 extra-nuclear functions even though the very recent discovery for a role of APE1/Ref-1 in RNA metabolism points to a potential function in the tumorigenic process [51,52]. ROS play a role in progression of CLD to HCC [9] and activates APE1/Ref-1. In fact, during cellular response to oxidative stress neo-synthesized APE1/Ref-1 is rapidly translocated into the nuclear compartment. Nuclear localization of APE1/Ref-1 is controlled by the first 20 amino acids at the N-terminus sequence through a nuclear localization signal (NLS) [49]. Qu demonstrated that S-nitrosation in response to NO stimulation leads to the nuclear to cytoplasmic APE1/Ref-1 translocation by a nuclear export signal [53,54]. Thus, both nuclear import and export may control sub-cellular distribution of APE,1 its intracellular trafficking and protein activities. It has been shown both that APE1/Ref-1 up-regulation is associated with an increased cell resistance toward oxidative stress [37] and that APE1-Ref-1 silencing enhances cell sensitivity to radiotherapy [55]. The reduction of APE1/Ref-1 mRNA and protein levels is associated to a reduced cell resistance to death [56]. Here we demonstrated that APE1/Ref-1 over-expression in hepatocytes confers cell survival advantage as an adaptive response to oxidative damage.

We observed that, in IHH, transfection with pRef-1 exert a significant protection both against H_2_O_2_ induced oxidative insult and against UV irradiation by reducing the hepatocyte apoptotic rate. It is known that APE1/Ref-1 knockdown sensitizes cells to apoptosis induced by oxidative stress both *in vitro* [57] and *in vivo* [58], that APE1/Ref-1 silencing increases HCC sensitivity to radiotherapy by enhancing hepatocyte apoptosis, nevertheless the mechanism directly involved in APE1/Ref-1 apoptotic control is not well understood.

We showed that APE1/Ref-1 over-expression reduce the hepatocyte apoptotic rate pointing to a possible link between APE1/Ref-1 over-expression and hepatocyte survival. Some pro-apoptotic molecules such as BAX are down regulated or inactivated in HCC [59] causing cell survival through mitochondrial apoptotic pathway dysfunction. [60]. Bhattacharyya demonstrated that APE1/Ref-1 expression reduce BAX production and apoptotic rate in Helicobacter pylori-mediated gastric epithelial cell apoptosis [61]. Mitochondrial targeted APE1/Ref-1 reduced the apoptotic Cytochrome C release [62] and absence of APE1/Ref-1 decreased the ratio of Bcl2/BAX protein expression resulting in Cytochrome C release and apoptosis [57]. In HCC the apoptotic rate is reduced, but, if BAX activation is induced, this apoptotic rate can be reversed [63] suggesting BAX as a potential therapeutic target in HCC. Interestingly, in our model APE1/Ref-1 over-expression was able to inhibit BAX activation and Cytochrome C release, and this is the first report demonstrating that APE1/Ref-1 over-expression may protect human hepatocytes from apoptosis acting at the mitochondrial apoptotic pathway level.

In summary, this study demonstrated for the first time that APE1/Ref-1 synthesis is up-regulated in human hepatocellular carcinoma and that mRNA level increases according to the progression of liver disease. We also showed that APE1/Ref-1 over-expression protects normal hepatocytes from oxidative stress damage and reduce apoptotic rate by inhibiting BAX activation suggesting another APE1/Ref-1 cytoplasmic role in hepatocyte survival. Although all these observation need to be confirmed in a larger cohort of patients, our findings point to APE1/Ref-1 as a promising molecular target for HCC diagnosis and treatment being implicated in hepatocyte survival and escape from apoptosis.
